# Therapeutics of the Throat

**Published:** 1888-01

**Authors:** E. B. Nash

**Affiliations:** Cortland, N. Y.


### THERAPEUTICS OF THE THROAT.

As previously mentioned in THE HOMŒOPATHIC PHYSICIAN, I have been arranging for a monograph on the therapeutics of the throat. Below is given a list of remedies, with names of physicians, whom I now earnestly beg to help me in the work. I hope each physician will try to work out the remedies given him, and so give practitioners a much needed assistance. I shall be glad to hear from the gentlemen whose names are given below. To succeed we must all work.

Yours fraternally,

E. B. NASH, CORTLAND, N. Y.

### REMEDIES AND WORKERS.

Acetic acid, J. T. Kent.
Aconite, E. J. Lee.
Aesculus, J. V. Allen (worked).
Ailanthus J. V. Allen (worked).
Alumina, M. Preston (pledged).
Amm.-carb., Amm.-mur., } W. M. James (pledged).
Anacardium,
Apis, W. P. Wesselhoeft (pledged).
Argentum, Argent.-nit. } E. Rushmore (pledged).
Arsenic, E. A. Ballard (pledged).
Arum tri., Wm. Jefferson Guernsey (worked).
Aurum, R. H. Bedell.
Baptisia, Geo. W. Sherbino (pledged).
Baryta,
Belladonna, W. S. Gee (published Nov., '87).
Borax, T. P. Birdsall.
Bryonia, W. H. Baker (worked).
Calcarea, Calc.-ph., } Franklin Powel.
Cantharis, C. Carleton Smith (pledged).
Capsicum, E. W. Sawyer (pledged).
Carbo an., Carbo veg. } Edward Cranch.
Causticum, George G. Gale.
Cistus, E J. Lee (worked).
Conium,
Creosote, Alice B. Campbell.
Digitalis,
Dioscorea, J. B. Bell (pledged).
Drosera, S. A. Kimball.
Dulcamara, A. McNeil.
Euphorbium,
Euphrasia,
Gelsemium, A. McNeil (worked).
Graphites, Samuel Long.
Gratiola,
Guaiacum,
Hepar, L. Hamilton Evans.
Hydrastis, Leila A. Ren Dell Goodrich.
Ignatia, G. H. Clark (worked).
Indium, J. B. Bell (pledged).
Iodium,
Kali bich., Kali carb., Kali-iod., } Clarence Willard Butler (pledged).
Kalmia,
Lac. caninum, E. W. Berridge.
Lachesis, Ad. Lippe (pledged).
Lachnanthes,
Lycopodium, P. P. Wells (pledged).
Magn.-carb., Magn.-mur., } J. B. Gregg Custis (pledged).
Manganum,
Mercurius, Merc.-bin. & prot. iod., } H. C. Allen, (pledged).
Mezereum,
Mur.-acid,
Natr.-ars., Natr.-m. & ph., }
Nitr.-acid, E. P. Hussey (pledged).
Nux-vomica, D. C. McLaren.
Petroleum, Julius Schmitt (pledged).
Phosphorus, Phosph -acid, } Wm. Jefferson Guernsey.
Phytolacca, E. B. Nash, published June, '87.

REMEDIES AND WORKERS—*Continued.*

Platinum,
Psorinum, W. A. Hawley (pledged).
Pulsatilla, J. A. Biegler (pledged).
Rhododendron,
Rhus-tox,
Sabadilla,
Sarsaparilla, Flora A. Waddell.
Senega,
Sepia, B. L. B. Baylies.
Silicea, John C. Robert.
Spongia, C. Carleton Smith.
Stannum, T. S. Keith.
Staphisagria, R. L. Thurston.
Strontia, J. W. Thomson.
Sulphur, J. T. Kent (pledged).
Sulph.-acid.
Thuja, J. E. Winans.
Veratrum,
Zinc, W. S. Gee.

Who will volunteer for remedies not having names set opposite them.

---

